# A Quality Improvement Initiative to Decrease Time to Analgesia in Patients With Sickle Cell and Vaso-Occlusive Crisis: A Population With Disparities in Treatment

**DOI:** 10.7759/cureus.29569

**Published:** 2022-09-25

**Authors:** Tyler Arnold, R. Lane Coffee, Leon Rosenberg, Seethal A Jacob, Sean Thompson, Heather Saavedra, Stephen John Cico, Brian Wagers

**Affiliations:** 1 Emergency Medicine, Indiana University School of Medicine, Indianapolis, USA; 2 Pediatrics, Indiana University School of Medicine, Indianapolis, USA; 3 Internal Medicine, University of Central Florida College of Medicine, Orlando, USA; 4 Clinical Sciences, University of Central Florida College of Medicine, Orlando, USA; 5 Office of Graduate Medical Education, University of Central Florida/Hospital Corporation of America (HCA) Florida Healthcare Graduate Medical Education (GME) Consortium, Orlando, USA

**Keywords:** disposition, analgesia, plan-do-study-act, quality improvement, sickle cell disease, vaso-occlusive crises

## Abstract

Introduction: Vaso-occlusive crises (VOCs) are the leading cause of emergency department (ED) visits and hospitalizations in patients with sickle cell disease (SCD). Timely administration of analgesia, within 60 minutes of patient registration, is the standard of care for SCD patients with VOCs. Patients with VOCs have longer times to initial analgesia compared to similar painful conditions. The primary aim of the project is to have 75% of patients with VOCs receive initial analgesia within 60 minutes of being registered, the current recommended time frame from the National Heart, Lung, and Blood Institute (NHLBI).

Methods: A multi-disciplinary team used quality improvement (QI) methodology to develop a plan involving multiple Plan-Do-Study-Act (PDSA) cycles. A rapid evaluation process was employed which included notification of a patient with a VOC being placed in a room, rapid evaluation by all team members and use of an electronic order set.

Results: The aim was met 72% of the time during our intervention period, compared to 17% pre-intervention. Average time to initial analgesia was decreased from 61 minutes to 42 minutes (p-value < 0.001), while time to disposition was also decreased when time goals were achieved.

Conclusion: Using a rapid evaluation process we were able to decrease time to initial analgesia in a patient population that has previously experienced delays in care and decrease overall time to disposition.

## Introduction

Sickle cell disease (SCD) is the most common inherited hematologic disorder in the United States, affecting over 100,000 Americans [1], with the African American population most affected [2]. A mutation in the β-globin gene leads to an abnormal hemoglobin variant causing sickling of red blood cells. This sickling leads to vaso-occlusion in the blood vessels, leading to complications such as severe pain. These painful episodes, known as vaso-occlusive crises (VOCs), begin as early as six months of age [3]. Roughly one-third of children with SCD experience a VOC by the age of one and almost all patients with SCD will experience a VOC by the age of four [3]. Pain is the leading cause of emergency department (ED) visits in patients with SCD [4].

A study has shown that SCD patients wait longer to be seen by a physician, as well as for initial analgesia, compared to similar painful conditions [5]. In addition, African American patients have delayed delivery of analgesia compared to the general population [5,6]. Delayed or inadequate treatment of VOCs can be associated with increased missed days of school or work, fear and mistrust in healthcare providers, and the development of chronic pain later in life [3,7]. It is important to understand that pain management in VOCs is guided by patient report of severity of pain [8,9]. While data suggesting that rapid administration of analgesia results in improved outcomes are mixed [10,11], best practice is to rapidly triage and administer analgesia to patients with SCD.

In 2014, the National Heart, Lung, and Blood Institute (NHLBI) published recommendations for the management of acute VOCs [12]. It is recommended the initial dose of analgesia be administered within 60 minutes of registration or within 30 minutes of triage of the patient [8,13,14]. A previously published study evaluating ED provider comfort and knowledge in managing acute sickle cell pain found that regardless of self-reported level of comfort in management, many providers were unaware of these NHLBI recommendations [15].

Intranasal fentanyl (INF) has previously been used as a rapid option for analgesia in many painful conditions. It is more potent than morphine with a high bioavailability [16], while the intranasal route leads to rapid delivery of the medication due to the highly vascularized nasal mucosa and direct connection to the central nervous system via the olfactory tissue [13]. In 2018, Fenster et al. demonstrated non-inferiority of INF compared to intravenous (IV) morphine in patients requiring incision and drainage of abscesses [16]. Specific to SCD, studies have shown INF to provide rapid pain relief with higher provider and patient satisfaction, as well as a decrease in pain scores with INF, compared to placebo in patients with VOCs [11,15]. Additionally, INF has been shown to decrease time to medication administration in a small study with a nurse-driven protocol [17] and retrospectively [13]. INF also provides an advantage in patients with SCD because obtaining IV access can be difficult due to scared veins secondary to their disease process and history of multiple IV sticks [11]. 

The primary aim of the quality improvement (QI) project was to have 75% of sickle cell patients with VOCs receive the first dose of either INF or IV analgesia within 60 minutes of registration using QI methodology, and in doing so, we also hoped to demonstrate decreased time to disposition.

## Materials and methods

This study was approved by the Indiana University Institutional Review Board as an exempt study. Riley Hospital for Children is a 379-bed tertiary care, free-standing pediatric hospital in Indianapolis, IN. The hospital is a level 1 pediatric trauma center with a 30-bed emergency department that sees over 52,000 patients per year. Providers in the emergency department include pediatric emergency medicine (PEM) physicians, general pediatricians, PEM fellows, residents (pediatrics, emergency medicine), and medical students. We utilized the SQUIRE (Standards for QUality Improvement Reporting Excellence) 2.0 guidelines to best design and execute this quality improvement study [18].

A multi-disciplinary team comprised of nurses, pharmacists, medical informaticists, pediatric hematology, and pediatric emergency medicine physicians determined barriers to meeting our goals using a key driver diagram. Data collection began in July 2018 prior to the first Plan-Do-Study-Act (PDSA) to determine the baseline time to analgesia and the route of administration of that medication prior to initiating the interventions. We used our electronic medical record (EMR) for data collection and included all sickle cell genotypes and patients under 18 years of age experiencing pain (International Classification of Diseases (ICD) 10 codes: D57.00, D57.01, D57.02, D57.211, D57.212, D57.219, D57.411, D57.419, D57.811, D57.819). ICD 10 codes for genotypes were confirmed by chart review. We excluded patients with acute chest, stroke, splenic sequestration, or fever.

**Figure 1:**
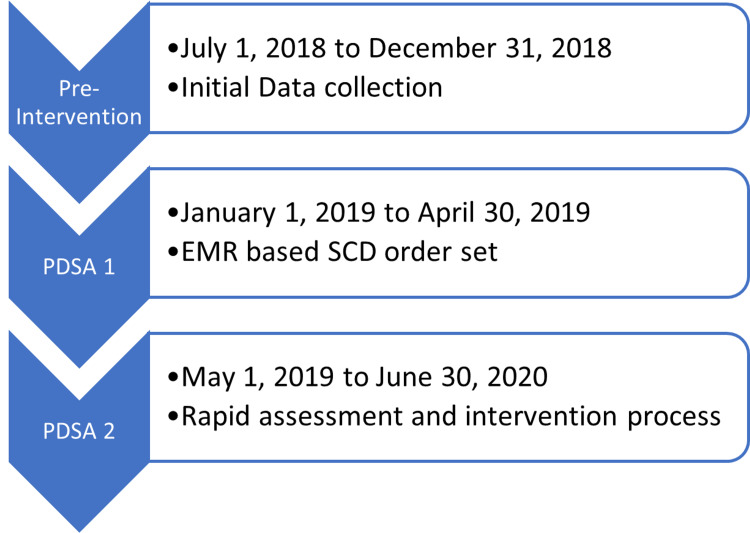
Flow diagram denoting the pre-intervention period through the second Plan-Do-Study-Act (PDSA) cycle

The pre-intervention period for which data was collected was July 1, 2018, through December 31, 2018. The first PDSA cycle involving the use of a SCD order in the EMR began January 1, 2019, and ran through April 30, 2019. The second PDSA cycle in which we attended daily nursing meetings and instituted a rapid assessment protocol began May 1, 2019, and ran through the end of the data collection on June 30, 2020, (Figure 1).

The initial intervention involved the formation of a “Sickle Cell Pain Management” order set within the EMR by this same multi-disciplinary team. This involved initial dose of medication, in addition to subsequent doses upon re-evaluation. It gave the providers a rapid method for ordering analgesia, providing multiple options, including intranasal fentanyl as the primary choice. It also allowed providers to order labs within the same order set.

The second PDSA cycle involved daily education and the initiation of a new process for rapid medication administration. The process began by the triage nurse using our ED all-call system to announce the room the patient with a VOC would be going to. We would then rapidly evaluate the patient as a team that included nursing, resident and/or fellow, and attending physician; patient care technicians and child life would be present for the initial assessment if in the ED. Traditionally the nurses would do their assessment and assign an Emergency Severity Index (ESI) triage level [19], followed by any learners before the attending physician would evaluate the patient. In the rapid evaluation, all involved persons would be notified by verbal notification and would go to the room immediately to perform an initial assessment. A brief history would be confirmed by all members of the team, a focused physical examination would be performed, and the first dose of analgesia would be ordered immediately in the patient room by the ordering physician (resident, fellow, or attending). This new process was discussed in daily nursing meetings for two weeks at the beginning of the second PDSA cycle. Physicians were encouraged to use both the order set and the new rapid evaluation process to quickly and rapidly order and deliver analgesia.

Each month we would evaluate all encounters for pain in patients with SCD and record the data points. We were evaluating each case for time to medication delivery, time to disposition, route of medication administration, age, sex, and SCD genotype. We then used standard t-tests in Microsoft Excel (Microsoft Corporation, Redmond, Washington) to determine statistical significance using individual group means. We used control charts to evaluate the data points for the percentage of patients meeting the goals and used run charts to evaluate the individual patients and time to medication administration.

## Results

Over the two-year study period, we analyzed 251 encounters with a primary or secondary diagnosis of SCD and a chief complaint of pain. Fifty-five encounters were pre-intervention and 196 encounters were post-intervention. The mean age was 14 years old. Females were 49.5%, 50.5% were males, and all were African American. One-hundred fifty-two encounters involved patients with hemoglobin (Hb) SS disease, 64 encounters with Hb SC disease, 21 encounters with Hb S-Beta 0 thalassemia, and 14 encounters with Hb S-Beta + thalassemia.

**Figure 2:**
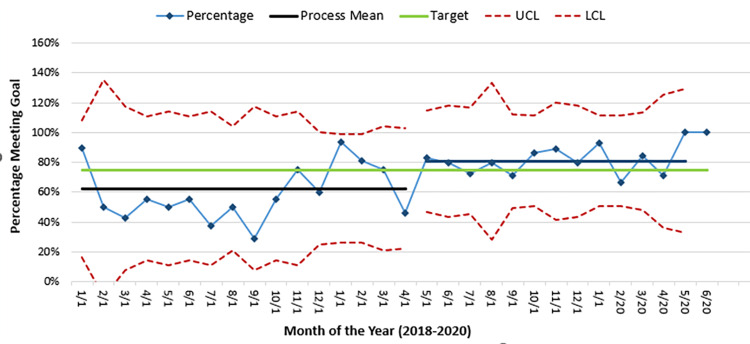
P chart denoting percentage of patients who met the study time goals by month UCL=Upper Confidence Limit; LCL=Lower Confidence Limit

The goal of 75% of patients receiving initial analgesia within 60 minutes from registration occurred in only one of the six (Jan-Jun) months leading up to the start of the initial PDSA cycle. During the intervention period, we achieved the goal in 13 of the 18 months (Figure 2).

**Figure 3:**
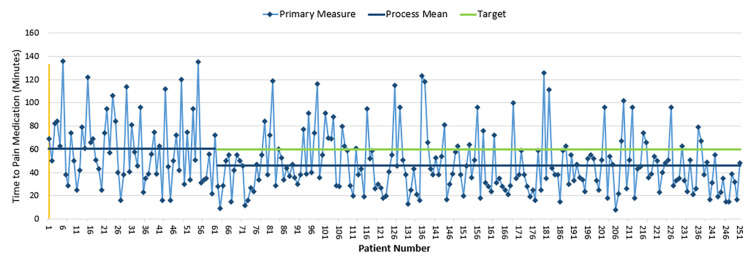
Time to first dose of analgesia from the patient registration

During the pre-intervention data collection, the average time to medication administration was 62 minutes. This was decreased to 44 minutes through the entirety of the second PDSA cycle (p-value < 0.001) (Figure 3). The overall average time to disposition pre-intervention was 267 minutes and post-intervention was 227 minutes (p-value: 0.011). When we met the goal of administering the medication within 60 minutes from registration, there was an average time to disposition of 227 minutes, while, if the goal time was not met, the average time to disposition was 260 minutes (p-value: 0.023).

During the entirety of the data collection, we found that the average time to first dose of intravenous medication was 61 minutes, while the average time to first dose of intranasal medication was 42 minutes (p-value < 0.001). We did not see a statistically significant difference in time to disposition when INF was used versus IV medication. In the pre-intervention group, the average time to disposition was shorter in the IV medication group (254 minutes versus 287 minutes, p-value: 0.32). After the first PDSA cycle, the average time to disposition based on the route of medication administration was 208 minutes pre-intervention vs. 225 minutes post-intervention (p-value: 0.43). After the second PDSA cycle, we did have a shorter average time to disposition in the INF group (230 minutes) versus the IV group (235 minutes), but this was not statistically significant (p-value: 0.79).

## Discussion

The goal of the project was for 75% of patients with SCD seen for VOC to receive an initial dose of analgesia within 60 minutes of registration. In the 18 months since starting the PDSA cycles, we were able to attain the goal in thirteen of the eighteen months (72%), where in the six months of the initial data collection, pre-intervention, that was only achieved once (17%). Additionally, we decreased the time to medication administration and time to disposition from the pre-intervention group to the post-intervention group. This demonstrates that the process of rapid team evaluation is effective for timely patient assessment and medication administration, regardless of which medication is used.

Previously, INF had been shown to decrease time to medication administration in a small study with a nurse-driven protocol [17]; however, this can be difficult to implement broadly, as many facilities, including our own, will not approve nurse-driven protocols for delivery of opiate analgesia. While we did see a shorter time to medication administration with INF versus IV medication in the study, we did not see a difference in time to disposition. We did, however, see a difference in time to disposition when patients received their initial analgesia within 60 minutes, which suggests that the process of rapid evaluation and medication administration is more important than the route of medication delivery. Kelly et al. retrospectively showed a similar decrease in time to analgesia [13]; however, it is unclear if any QI methodology was used in their processes.

Pain management in VOC, as recommended by the NHLBI, as well as the American Society of Hematology and the American Academy of Pain Medicine, should be guided by patient report of severity [8,9]. However, a study has shown that healthcare providers are more likely to perceive patients with SCD as drug-seeking or opioid-dependent, which results in inadequate treatment and more suffering [20]. While data suggesting that rapid administration of analgesia results in improved outcomes are mixed, the best practice is to rapidly triage and administer analgesia to patients with SCD. It is crucial to treat self-reported pain in a timely manner to avoid fear and mistrust in healthcare providers and the development of chronic pain later in life. We show here that with appropriate rapid evaluation and medication administration, regardless of the medication, we can more quickly and efficiently treat and disposition a population that previously has had disparities in management.

Study limitations include the lack of data related to time from triage. In the NHLBI guidelines, the recommendation is for sixty minutes from registration or thirty minutes from triage. However, using our EMR, it is difficult to track the triage time as there is no definitive marker for this. Therefore, in the analysis, we decided to use the time from registration as we knew this would be a more reliable method when reviewing our patient encounters. One of the other difficulties we encountered was with nursing turnover. National ED nursing turnover is 14% [21] and during 2019 we had 35% nursing turnover. Despite this turnover rate, we were successful with the initiative, suggesting this is a viable intervention to improve analgesia time for patients with SCD. At our hospital, we do use similar alerts for other painful processes, such as long bone fractures, so it allowed for adaptability for our patients with SCD, which may have aided in our success despite higher turnover. Additionally, this was a single-center study and further studies would need to be done to see if this is generalizable on a larger scale. Any patient with a history of SCD underwent rapid assessment team evaluation regardless of age, pain severity, time of day, or other confounding factors.

## Conclusions

Using QI methodology, we were able to increase the percent of patients meeting the NHLBI recommendations for first dose of analgesia within 60 minutes of triage for patients presenting with VOC in a majority of the months during the post-intervention period. This represents a statistically significant increase from the pre-intervention period. The greatest improvement was seen following the initiation of our rapid evaluation process. Furthermore, we showed improved time to analgesia in patients with VOCs and significantly decreased time to disposition regardless of route of medication administration. This was unique to this study as other similar studies showed their results exclusively with the intranasal route of administration and unrelated to time to disposition. Other EDs may be able to utilize similar QI methodology to improve the care of patients with VOC and meet the NHLBI recommendations.
